# Person-centred study on higher-order interactions between students’ motivational beliefs and metacognitive self-regulation: Links with school language achievement

**DOI:** 10.1371/journal.pone.0289367

**Published:** 2023-10-04

**Authors:** Ioannis Katsantonis, Ros McLellan

**Affiliations:** Faculty of Education, University of Cambridge, Cambridge, United Kingdom; The University of Auckland, NEW ZEALAND

## Abstract

Variable-centred studies assume that the links between motivation and metacognition with academic achievement are uniform across all students. However, this assumption may not hold and multiple interactions between motivational beliefs and metacognitive self-regulation may occur. To this end, the present study sought to explore these higher-order interactions and their links with school language achievement in a low-performance context. A large sample (N = 1046, 53.14% girls) of Greek secondary school students (*M* = 13.97, *SD* = .80) was drawn. Latent profile analyses were deployed to mimic higher-order interactions. Unexpectedly, the results indicated only three distinct well-defined profiles of students’ motivated metacognitive self-regulation, namely exceptional motivation and metacognitive self-regulation (23.3%), adequate motivation and metacognitive self-regulation (48.2%), and minimal motivation and metacognitive self-regulation (28.5%). Incompatible profiles of motivation and metacognitive self-regulation did not emerge, contrary to previous findings suggesting negative higher-order interactions. The BCH method revealed large mean differences in school language achievement between the profiles, adjusting for covariates. Latent multinomial logistic regression indicated that gender and age predicted greater odds of membership to the minimal motivation and metacognitive self-regulation profile. Socio-economic status and spoken language at home predicted less chances of membership to the minimal profile only. Educational interventions are needed to target both motivational beliefs and metacognition to prevent underachievement.

## Introduction

Motivation and metacognition have been posited as important components of students’ self-regulated learning (SRL) [1–3] and have been linked with adaptive student outcomes, such as achievement [4–7]. However, extant studies make an assumption that may not be tenable; that is, they take a variable-centred approach and assume that the links between students’ motivational beliefs and application of metacognitive strategies are uniform across all sample members [8,9]. In other words, these studies estimate “averaged” separate effects of motivation and metacognition on achievement. In reality though, students in a sample may constitute a heterogenous population and demonstrate different levels of motivation and metacognition that are connected differentially with achievement. However, profiling studies of motivation and metacognitive self-regulation are inconclusive. To address this important gap in extant research, we can deploy a person-centred latent profile analysis that assigns students in groups with similar characteristics and is able to mimic higher-order interactions [10,11] between multiple motivational variables and metacognitive self-regulation.

Some person-centred studies have explored heterogeneity in students’ motivational and metacognitive characteristics and potential connections with achievement [12–14]. However, most of these studies use university/college samples. Consequently, their findings do not reflect the motivational and metacognitive processes that take place in secondary school classrooms during adolescence when metacognitive abilities are malleable and subject to developmental spurt [15] and academic motivation usually declines [16–18]. Additionally, extant studies were conducted in high-performance contexts (e.g., USA, Germany, China, etc.), as defined by PISA rankings [19], and do not illustrate what occurs in low-performance contexts, such as the Greek one [20] that is the focus of the present study. Finally, there exist studies that explore secondary school students’ motivational or metacognitive strategies separately [21,22], whereas we need to consider both aspects simultaneously to identify potential areas for theoretical and practical educational innovations.

Hence, the present study sought to address these important gaps in the literature by exploring unobserved heterogeneity and higher-order interactions between motivational beliefs and metacognitive strategies of adolescent secondary school students.

### Interactions between motivation and metacognition in SRL

Current models of SRL [1–3,23] integrate both metacognitive processes and academic motivational beliefs and postulate that learners need to activate both in order to be academically successful. Metacognition is broadly defined as “cognition about cognition” and involves cognitive processes that take place on a “meta-level” requiring monitoring and control of cognition [24]. In the present study, the focus is specifically on metacognitive self-regulation which is defined as the regulation of cognitive processing (i.e., planning, monitoring, and regulating strategies) instead of metacognitive knowledge [25] since metacognitive self-regulation is considered a higher-order process that reflects students’ ongoing processing and strategies deployed to improve performance, rather than static general knowledge about the person, task, and strategies [26]. Motivation is broadly defined as the forces that make people act and academic motivation comprises self-beliefs of competence (e.g., self-efficacy), goals (e.g., goal orientations), and intrinsic (e.g., interest) and extrinsic (e.g., incentives) motivation, amongst others [27].

Empirical research has indicated how critical motivation is for metacognition [6,28]. Additionally, other researchers have specified both motivation and metacognition as joint predictors of students’ performance [29–32]. Yet, these studies have not captured the complexity of SRL since they have not tested the multiplicative effects of motivational beliefs with metacognition. As Efklides et al. (38) accurately mention, SRL is not only informed by the recursive prediction from motivation and metacognition but also by the interactions between these components. This study adopts Efklides’ [1] MASRL- Metacognitive and Affective Model of SRL- which takes an interactionist perspective. The MASRL is structured at two levels, namely the person-level and the task X person level [33]. The person-level is more generic and encompasses the interactions between students’ relatively trait-like cognitive, metacognitive, motivational, and emotional characteristics, whereas the task X person level involves cognitive, emotional, and motivational regulation while engaging with a task [34]. At each level of the model, it is hypothesised that the postulated components would interact [1]. The present study focuses only on the motivational and metacognitive self-regulatory aspect at the person-level. However, the directional nature of these interactions is under-researched.

Students’ motivational beliefs positively correlate [35–37] indicating that students hold multiple motivational beliefs at the same time. Thus, the order of possible interactive effects of motivation with metacognitive self-regulation becomes large and difficult to estimate [38]. Therefore, the present study contributes to theoretical models of SRL by deploying a person-centred latent profile model to explore the higher-order interaction of motivation and metacognitive self-regulation (i.e., self-efficacy X mastery goal X performance goal X intrinsic motivation X extrinsic motivation X metacognitive self-regulation) and to link this with school language achievement.

### Related profiling approaches to the study of motivation and metacognitive strategy use

Several studies have explored the interactions of students’ metacognitive self-regulation in terms of metacognitive strategy use and motivational factors. These studies use mostly person-centred approaches to identify profiles of students’ motivational and metacognitive self-regulatory characteristics. Person-centred approaches, such as latent profile analysis, do not accommodate interaction effects in the same ways as conventional regression modelling. Nevertheless, the distinct subgroups/profiles of students’ characteristics mimic higher-order interactions that would have been impossible to disentangle otherwise using regression modelling [10,11,39]. Therefore, person-centred evidence can be interpreted in terms of both heterogenous profiles of and complex interactions between motivational beliefs and metacognitive self-regulation.

Studies having deployed person-centred (i.e., latent profile/ cluster analyses) approaches indicate the possibility of encountering more than three subgroups of students’ motivational and metacognitive self-regulation. Yet, conclusive results cannot be reached since each study offers new insights. Research studies using school-aged student samples have also revealed various emerging profiles. For instance, a study revealed four profiles of motivational and metacognitive self-regulation characteristics ranging from unmotivated learners to maximal learners [13]. Another study explored profiles of motivation and metacognitive strategy use of second language learners and found four profiles of students that were characterised by varying scores on motivational and strategy variables [12].

In the same vein, studies with college/university samples have illustrated significant variation of emerging profiles. For instance, studies have indicated five [40,41] or four [42] profiles. The emerging profiles included a mixture of different characteristics of motivation, metacognitive and cognitive self-regulation with some studies suggesting the existence of both positive and negative interactions. However, a research gap is that all the above studies did not examine the links between the profiles with students’ native language attainment, but focused on specific grades in certain university/college samples. Finally, most studies with university samples had small-to-medium sample sizes, which may be underpowered for profile analyses [43].

There is also preceding evidence that profiled students’ motivational [22,44] and (meta-)cognitive self-regulatory strategies [21,45] separately. However, such profiling evidence is limiting since it does not permit study of how different aspects of academic motivation and metacognitive self-regulation interact in the prediction of language achievement. To this end, the present study sought to address this limitation by including both a range of motivational indicators and metacognitive self-regulation in the profiling to predict school language achievement.

What is interesting, though, is that both secondary school and university studies identified some profiles where students scored higher on motivation but lower on certain metacognitive or cognitive strategies. This suggests that the potential interactions between students’ motivational beliefs and metacognitive self-regulation may be more complicated than initially thought, namely they may be negative. Since the MASRL model does not specify the sign of the interactions [1,46], more research is needed on the complex interactions between motivational beliefs and metacognition to better understand the SRL processes at the general person-level.

### School language achievement: A critical outcome

Language is such a necessary ingredient for human communication since our mental representations, learning, and cognition rely heavily on language [47,48]. Young students live in a swiftly changing world where the demands to understand and produce a quantity and variety of written materials are increasing and becoming more complicated [49]. Given these new challenges that students face, they require to acquire new cognitive and metacognitive competencies in order to process linguistic inputs [49]. However, most of the studies profiling students’ motivational and metacognitive self-regulation characteristics have not connected these profiles/ interactions with school first language (L1) attainment, which captures a range of linguistic skills (e.g., abstract writing, text comprehension, essay writing, reading literacy, etc.) that are taught in the secondary school in Greece (Law 21072b/C2). This is a significant gap in the literature that the present study seeks to address.

### The nature of the Greek language subject and the educational system

The Greek language subject in the Greek secondary schools is usually taught by specialised language teachers that have completed four-years university education in linguistics/ philology. The educational system is centralised and all students at the same year-groups are taught using the same language textbooks (that are centrally provided) based on the common national curriculum for Greek modern language [20]. The Greek modern language subject is taught between two and three hours per week in the junior high school (Gymnasium) [50] and two to four hours in senior high school (lyceum) [51]. According to official guidelines for teaching the Greek modern language, the main aims are to enhance the linguistic and communicative capabilities of students through reading and comprehending written and oral speech, the development of critical thinking, the understanding of grammatical phenomena, the recognition of text genres, and production of written texts in authentic communication frameworks [50]. Given the above, students’ school language achievement reflects to what extent students had acquired a range of competencies. However, according to PISA 2018 findings, more than 25% of the Greek adolescent students (aged 15 years old) had achieved below the “minimum level of proficiency” that all students should achieve by the end of secondary education, as outlined in the United Nations Sustainable Development Goals [19]. Therefore, it is more crucial to conduct research in this low-performance setting to understand the reasons that drive students’ achievement and underachievement.

### The present study

It has become clear that most of reviewed studies were conducted in high-performance countries, with small-to-medium sample sizes, and using university/college student samples. A smaller proportion of extant studies utilised second-language learners. All these facts pose some theoretical and practical limitations in terms of developmental sensitivity and educational value. Hence, it is important to address these limitations by including a range of motivational beliefs (e.g., self-efficacy, mastery and performance goal orientations, intrinsic and extrinsic motivation) in the profiling of metacognitive self-regulation of secondary school students and to link these profiles with school language achievement. To address the potential issue of confounding, we also included a few socio-demographic variable as covariates based on preceding evidence. These covariates were the following. Firstly, gender was included as a covariate since gender differences have been noted in both motivation and metacognition [6]. Secondly, students’ socio-economic status (SES) was also included since it has been associated with achievement gaps [52], but is seldomly included as a predictor of students’ motivational and metacognitive profiles. Thirdly, students’ age was also accounted for given that greater age has been associated with better metacognition [15] but lower academic motivation [16]. Finally, whether students spoke Greek at home (as a proxy of non-native language learners) with their families was also controlled since it has been found to influence students’ achievement [53]. Overall, to guide this study, the following research questions and hypotheses were formulated:

RQ1: How do motivational and metacognitive characteristics co-occur in the same students? How are the interactions between motivational and metacognitive characteristics formed?

RQ2: How do different profiles of common motivational and metacognitive characteristics influence school language achievement?

RQ3: How are the covariates (socio-economic status, sex, age, and (main) language spoken at home) associated with the profiles of motivation and metacognitive self-regulation?

It is hypothesised that more than three profiles of students’ motivational and metacognitive self-regulatory characteristics would emerge (H1). It is expected that one profile would include interactions between high motivation and high metacognitive self-regulation (H2), whilst another profile would include interactions between low motivation and low metacognitive self-regulation (H3). It is possible that another profile including a negative interaction would occur such as low motivation and high metacognitive self-regulation, or the reverse (H4).It is expected that the emergent interactions through the person-centred approach would be linked differentially with school language achievement (H5). Additionally, identifying as a male would be associated with membership to the less motivated and metacognitive self-regulation profiles (H6). Further, we hypothesised that students from higher SES backgrounds would be more motivated and display greater metacognitive self-regulation (H7). Given the ambiguity regarding the association of age (i.e., a proxy of developmental level) with motivation and metacognitive self-regulation profiles, we did not formulate a specific directional hypothesis but only assumed that an association would emerge (H8). Finally, it was expected that speaking Greek at home would be associated with greater likelihood of membership to the more motivated and metacognitively self-regulated profiles since native speakers would probably face less difficulties in the school language lesson (H9).

## Materials and method

### Participants

The sample of the present study comprise 1,046 Greek secondary school students (*M*_age_ = 13.97, *SD* = .80) from 19 schools. The data collection took place between late 2022 and late April 2023. The sample consisted of 46.86% boys and 53.14% girls. Students come from a range of socio-economic backgrounds but overall were of average SES (*M* = 6.38, *SD* = 1.6). The majority of the students spoke Greek at home (94.42%), and were sampled from urban (N = 12), semi-urban (N = 4), and a few rural areas (N = 3). The above demographics are aligned with population estimates according to national averages [54]. The present sample is situated within the developmental stage of adolescence, when significant biological (e.g., puberty, brain development), educational (e.g., transitions from primary to secondary school), and relational (e.g., romantic relationships) changes occur [55]. Thus, the present sample’s developmental characteristics differ from those of previous studies with college students.

### Measures

Several scales from the Motivated Strategies for Learning Questionnaire (MSLQ) [56,57] were deemed appropriate for adolescents aged ~14 years old and were administered. Additionally, the MSLQ has been utilised in a plethora of studies, translated to different languages (including Greek- [30,58,59], and administered to various populations in different educational and cultural contexts [60]. All MSLQ items are scored using a 7-point rating scale ranging from 1 “not at all true of me” to 7 “very true of me” [57]. The question prompts were adapted to refer to the modern Greek language lessons that students are taught as part of the national curriculum. Item wordings and coding decisions are presented in supplementary materials.

#### Self-efficacy

The scale comprises 9 items (see Table in S1 Table) measuring perceptions of competence and performance in the language class [57]. Reliability coefficient indicated exceptional reliability, Cronbach’s α = .91.

#### Mastery and performance goal orientations

Two scales were tapping into mastery and performance goals. The *mastery goal orientation* scale comprises 4 items (see Table in S2 Table) measuring students’ perceptions of the reasons why they engage with the lesson, such as developing competence, curiosity, and challenge [56]. The reliability coefficient indicated adequate reliability, Cronbach’s α = .68. The *performance goal orientation* scale consists of 4 items (see Table in S3 Table) that measure students’ perceptions of engagement in the modern Greek language lesson for reasons of displaying competence, rewards, and outperforming others [56]. The reliability coefficient indicated good reliability, Cronbach’s α = .78.

#### Intrinsic motivation and extrinsic motives

The 6-item long task value scale was split into two separate scores because a confirmatory factor analysis (CFA) rejected the unidimensional structure. However, a CFA (see results section) confirmed the two-factor structure. Two items that described interest and liking the topics and content of the class (i.e., emotional aspect) were assumed to reflect intrinsic motivation (see Table in S4 Table). Cronbach’s α = .82 indicated good reliability for the intrinsic motivation scale. The other four items that described utility, valuing, and importance of the language class content were assumed to capture the extrinsic motivation aspect (see Table in S5 Table), in line with self-determination theory [61]. Cronbach’s was α = .86, demonstrating a good level of reliability.

#### Metacognitive self-regulation

The metacognitive component covers the control and self-regulatory part of metacognition [25]. Metacognitive self-regulatory strategies in this measure include planning, monitoring, and regulating [60]. Planning is operationalised as goal-setting, whilst monitoring is measured using indicators of tracking one’s task-/goal-relevant attention [56]. Finally, correcting one’s cognitive activities is used as an index of metacognitive control [56]. From the original nine items comprising the metacognitive self-regulation, three loaded on the factor very weakly even after reverse-scoring them due to negative content and were, thus, dropped from the scale (see Table in S6 Table). Cronbach’s coefficient of reliability indicated that the scale was reliable, α = .77.

#### Modern Greek language achievement

Grades in modern Greek language classes were reported by students. Studies have shown that self-reported grades are very reliable [62–64]. The Greek grading system in secondary education is numerical and ranges from one to twenty. Thus, grades in modern Greek language class potentially ranging from one to twenty were utilised.

#### Covariates

A few demographics were collected as covariates based on preceding research suggesting appreciable associations between these covariates, motivation, and metacognition.

*Gender*. A binary variable was used to code students’ gender as male versus female.

*Socio-economic status (SES)*. In this study, the families’ SES was measured using the youth version of the *MacArthur* scale [65], which is a ladder where students mark where they think their families are standing in the society. The possible values can range from 1 “lowest SES” to 10 “highest SES”.

*Age*. Students’ age was coded using an ordinal variable ranging from 13 to 16. Age was controlled.

*Language spoken at home*. A binary variable (1 = yes, 0 = no) was used to code whether students spoke Greek with their family at home.

*Current school grade*. Students’ current school grade was measured using an ordinal variable reflecting which grade students were studying in and was ranging from 0 “A Gymnasium” to 3 “A Lyceum”. We did not include this variable in the main analyses as a control variable because of overlap with students’ age and due to very possible confounding.

### Statistical analyses

Confirmatory factor analyses (CFA) were estimated per scale to explore the internal structure validity of the scales [66]. A weighted composite score was derived per scale by multiplying each item by its factor loading [67]. Afterwards, the six weighted composite variables were z-scored (M = 0, SD = 1) so that they were placed on the same metric and were more comparable [68]. Latent profile analyses (LPA) were estimated based on the z-scored variables. LPA is a probability model that assigns participants into k latent profiles that share similar characteristics on the measures [69]. The LPA model, unlike cluster analysis, allows for a mathematical evaluation of how well each LPA solution fits the data and accounts for both measurement and classification error [43].

**Fig 1:**
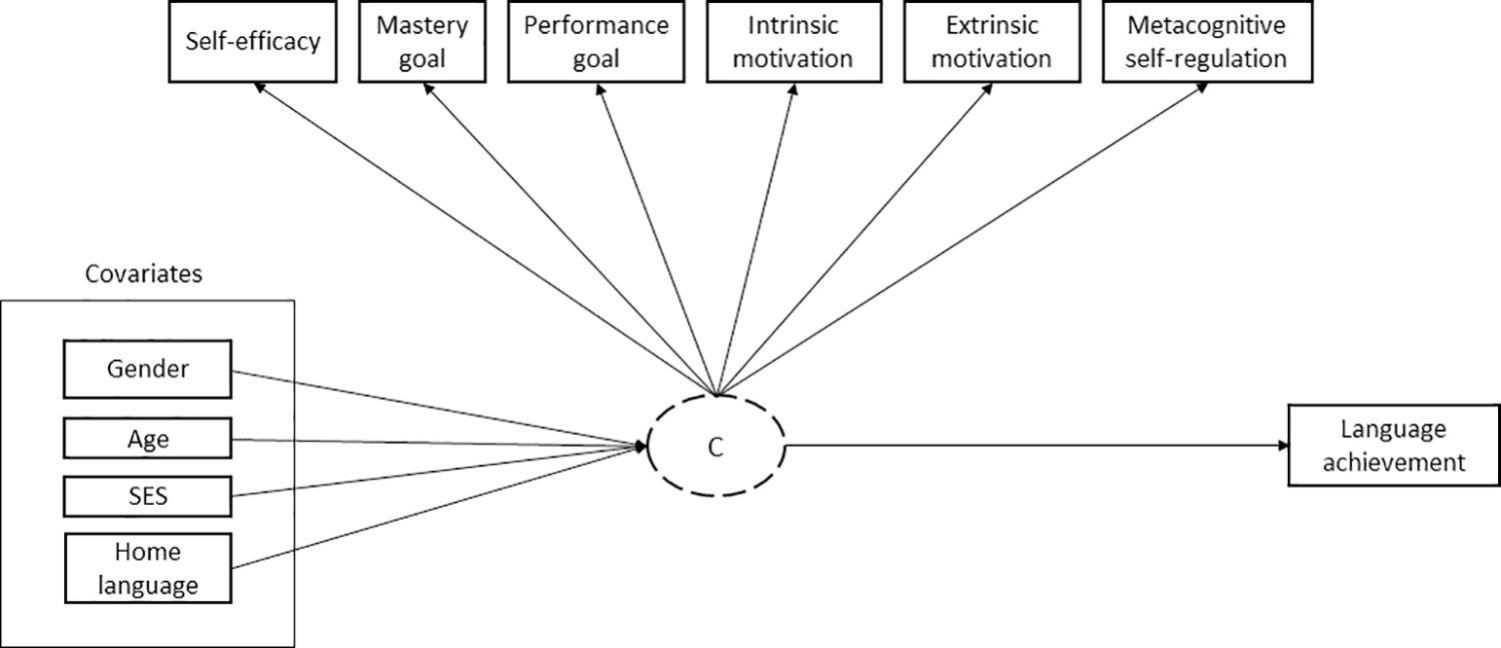
Categorical latent variable predicting language achievement conditionally on covariates.

To examine the links of profile memberships (including the interactions) with school language achievement, the manual BCH method was deployed since it does not shift the number of latent profiles by including more indicators and can, additionally, accommodate relevant covariates [70,71]. Given a fraction of missing data on covariates, a multiple imputation was conducted in M*plus* [72] using all the variables above since full-information maximum likelihood is not available with the BCH method [70]. The full conceptual model is presented in Fig 1.

The small sample size at the school-level (N = 19), did not justify multilevel modelling or cluster-robust standard errors since the estimated parameters exceeded the number of clusters [73]. However, intra-class correlation coefficients (ICC) were derived from variance components (no-predictor random-intercept) models to explore the extent to which nesting of students within schools had to be taken into account using alternative approaches. An ICC below .05 would indicate that a multilevel modelling will have few benefits and be unreliable to estimate [74]. In case of an ICC greater than .05, a fixed-effects estimator was chosen to adjust the standard errors and account for unobserved school-level covariates [75,76]. This was achieved by regressing the achievement and the latent categorical variables on 18 dummy variables (19 schools minus 1).

To evaluate how well a proposed CFA model represents the data, the conventional fit indices were utilised. Specifically, CFI and TLI values close to/above .95 along with RMSEA and SRMR values below .06 and .08, respectively, were considered indicators of exceptional fit of the model to the data [77]. Factor score determinacy (FDET), defined as the correlation between the true and estimated factor scores, was also calculated. FDET is an indicator of the uncertainty of the latent factor measurement [78] with values close to 1 indicating less uncertainty and better measurement [79].

For profile enumeration in LPA, multiple fit indices were considered along with the practical interpretability of the profile solutions [80,81]. The Bayesian (BIC) and the sample-adjusted Bayesian information criteria (a-BIC) were evaluated along with the Vuong-Lo-Mendell-Rubin (VLMR), the Lo-Mendell-Rubin (LMR), and bootstrapped (BLRT) likelihood ratio tests that compare the model with the n-1 profiles with the model with n profiles. It is known that the information criteria can keep decreasing with additional profile solutions [43]. Similarly, the likelihood ratio tests can reach statistical significance in the presence of a large sample size [80]. Therefore, the ‘elbow’ plot of the information criteria values can be used to identify the point of diminishing returns similar to a variance explained measure as additional profiles are extracted [69,82]. Additionally, the classification quality was also considered, whereby entropy (E) values above .8 are preferable [69].

All main statistical analyses were performed using the M*plus* 8.7 software [79], whilst preliminary data management was performed using Stata 16 [83]. Since the BCH method does not provide effect sizes, Cohen’s *d* was calculated using Lakens’ (2013) calculator, whereby values equal to .2 are small, values equal to .5 are medium, and values equal to/above .8 are large [84]. Partial eta squared (η^2^) effect sizes were also calculated for between-profile differences, whereby values equal to .01 are small, .06 are medium, and .14 are large [84].

### Procedure

This study’s data collection protocols have received ethical approval by the Psychology and Education Research Ethics committee at the Faculty of Education, University of Cambridge, UK (29/7/2022). Parents/legal guardians of the students provided written informed consent and the students assented to participate. The questionnaires were administered by the lead researcher in schools. Permission to conduct the research in schools was granted by the Greek Ministry of Education (REF: 145640/Δ2/23-11-2022). The authors have no access to information that could identify individual participants during or after data collection.

## Results

### Preliminary analyses

Descriptive statistics and correlations are presented in Table 1. As seen in Table 1, students simultaneously hold multiple motivational beliefs since the correlations were all positive between the motivational beliefs. Outlier and missing data analyses are presented in the supplementary materials (Text in S1 Text). The intra-class correlation coefficients (ICC) revealed that a multilevel modelling was not justified given the small between-school heterogeneity in motivational beliefs and metacognitive self-regulation. The only exception to this was school language achievement, whereby 15% of school language achievement was attributable to school-level differences.

**Table 1:** Descriptive statistics and bivariate correlations between covariates and outcomes.

Variable	1	2	3	4	5	6	7	8	9	10	11	12
1. GENDER	1											
2. LANG	-.02	1										
3. SES	-.02	-.01	1									
4. AGE	.00	-.04	-.09*	1								
5. CLASS	-.07*	-.02	-.09*	.74***	1							
6. SELF	-.14***	.16***	.21***	-.10***	-.08**	1						
7. MASTERY	-.17***	.13***	.10***	-.06	-.04	.54***	1					
8. PERFORM	-.02	.06	.08*	-.15***	-.14***	.28***	.22***	1				
9. INT	-.14***	.09**	.08*	-.13***	-.10***	.47***	.54***	.25***	1			
10. EXT	-.20***	.12***	.08*	-.16***	-.13***	.57***	.61***	.32***	.78***	1		
11. MCOG	-.16***	.11***	.10***	-.14***	-.14***	.54***	.52***	.31***	.53***	.62***	1	
12. GRADE	-0.18***	.06*	.10***	-.09***	-.08**	.54***	.26***	.06	.08***	.28***	.35***	1
Descriptive statistics												
*Min-Max*	0–1	0–1	1–10	12–16	0–3	6.52–45.63	2.24–15.69	2.56–17.89	1.67–11.69	3.12–21.86	3.55–24.84	2–20
*% Max*	46.86	94.42	4.4	1.25	1.63	.39	1.44	6.41	5.9	5.23	4.23	5.14
*M (SD)*	-	-	6.38 (1.60)	13.97 (.80)	1.45 (.69)	32.03 (7.61)	10.75 (2.80)	13.10 (3.43)	7.22 (2.73)	14.76 (4.56)	15.09 (4.82)	15.97 (2.86)
*ICC*	-	-	-	-	-	.02	.02	.04	.03	.04	.03	.15

Note: GENDER: Male; LANG: Whether student speaks Greek at home; SES: MacArthur socio-economic status scale; AGE: The numerical age of the students; CLASS: The school grade currently studying in; SELF: Academic self-efficacy in language lesson; MASTERY: Mastery goal orientation; PERFORM: Performance goal orientation; INT: Intrinsic motivation (interest and enjoyment); EXT: Extrinsic motivation (utility and value); MCOG: Metacognitive self-regulation; GRADE: School language lesson grade; ICC: Intra-class correlation coefficient; ***p < .001; **p < .01; *p < .05.

### Construct validity testing

As seen in Table 2, CFA indicated good construct validity for the scales following some minor modifications by introducing residual correlations according to the modification indices. The parameter estimates of the CFA models are presented in the supplementary materials in S7–S11 Tables.

**Table 2:** Fit indices for internal structure validity analyses (CFA).

Scale	Χ^2^(df)	CFI	TLI	RMSEA	SRMR	FDET
Self-efficacy	123.768 (27)***	.960	.947	.059	.032	.956
Mastery goal orientation	.820 (1)	1.00	1.00	.000	.006	.842
Performance goal orientation	6.917 (2)*	.991	.973	.049	.016	.867
Intrinsic & Extrinsic motivation^†^	44.285 (8)***	.980	.962	.066	.023	.936^a^; .948^b^
Metacognitive self-regulation	25.642 (8) **	.984	.970	.046	.021	.882

Note: † Two-factor model for intrinsic and extrinsic motivation; FDET: Factor score determinacy; a: FDET determinacy for intrinsic motivation; b: FDET determinacy for extrinsic motivation; ***p < .001; **p < .01; *p < .05.

### Exploring complex interactions between motivational beliefs and metacognitive self-regulation: A latent profile analysis

To begin with the analyses, a likelihood ratio test was calculated based on the two-profile solution to estimate whether the latent profile indicator variances should be freely estimated across latent profiles or constrained to equal. The LRT(6) = 176.99, *p* = .001, indicated that the latent profile indicators’ variances should be freely estimated across profiles. Hence, the LPA models were estimated with unequal indicator variances and means. The fit indices for the LPA modelling are presented in Table 3.

**Table 3:** Fit indices for LPA (unequal variances).

Np	Log-Lik	BIC	a-BIC	E	VLMR *p*	LMR *p*	BLRT *p*
2	-7750.005	15632.112	15571.765	.824	< .001	< .001	< .001
*2* ^*A*^	*-7656*.*136*	*15486*.*091*	*15406*.*688*	.*848*	*<* .*001*	*<* .*001*	*<* .*001*
**3** ^**A**^	**-7341.014**	**14946.232**	**14825.539**	**.827**	**< .01**	**< .01**	**< .001**
4^A^	-7212.588	14779.765	14617.782	.812	>.05	>.05	< .001
5^A^	-7072.721	14590.416	14387.143	.854	< .01	< .01	< .001

A: Unequal indicator variances across profiles; STARTS = 500 125 10; Np: Number of extracted profiles; Log-Lik: Loglikelihood; BIC: Bayesian Information Criterion; a-BIC: Sample-adjusted BIC; E: Entropy; VLMR: Vuong-Lo-Mendell-Rubin LRT test; LMR: Lo-Mendell-Rubin LRT test; BLRT: Bootstrapped LRT.

**Fig 2:**
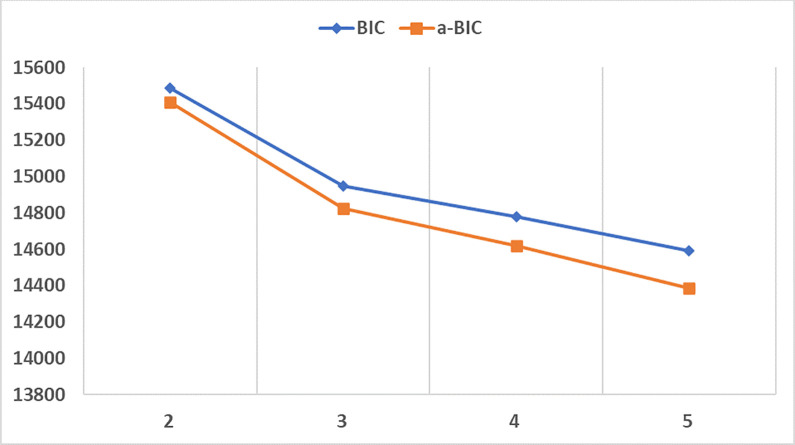
‘Elbow’ plot of BIC and a-BIC values for LPA models with 2 to 5 profiles.

From the (a-)BIC values in Table 3, it became clear that more than five profiles should be extracted. However, the VLMR and LMR LRT were in favour of the three-profile solution since the four-profile solution did not differ significantly from the three-profile solution. Given the inconsistent fit indices, the (a-)BIC values were plotted as shown in Fig 2. The ‘elbow’ plot indicated that the point of diminishing returns is reached at three profiles. Further profiles were also extracted but they did not represent theoretically meaningful solutions since they simply splintered the already well-defined three-profile solution. Therefore, the three-profile solution was retained.

**Fig 3:**
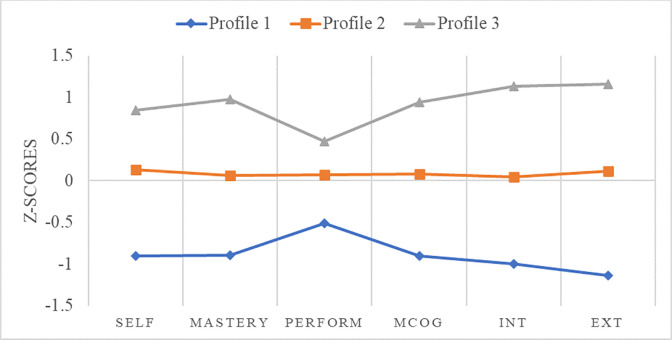
Plot of LPA indicator means; Profile 1: Minimal MMSR (28.5%); Profile 2: Adequate MMSR (48.2%); Profile 3: Exceptional MMSR (23.3%).

LPA latent indicator means were plotted (Fig 3) to understand how the variables interacted within and across profiles. It appeared that motivational beliefs went hand-in-hand with metacognitive self-regulation. The analysis showed that the students *within* each profile had similar levels of motivation and metacognitive self-regulation, whilst profiles where students scored high on motivation but low on metacognitive self-regulation, or the reverse, did not emerge. Thus, it was assumed that the sixth-order interaction between self-efficacy beliefs, mastery and performance goal orientations, intrinsic and extrinsic motivation, and metacognitive self-regulation was positive.

A significant proportion (28.5%) of the students belonged to profile 1 that was characterised by extremely low scores on motivational beliefs and metacognitive self-regulation, and was, thus, called “*minimally motivated and metacognitively self-regulated students*” (minimal MMSR). Consequently, these students seemed to have extremely low capability to self-regulate their learning. Only 23.3% of the students were scoring nearly 1 SD above the mean of the sample and were named “*exceptionally motivated and metacognitively self-regulated students*” (exceptional MMSR). These students were expected to be extremely proficient in self-regulating their learning. Finally, the majority (48.2%) of the students appeared to score on the mean of the motivational beliefs and the metacognitive self-regulation scales and were labelled “*adequately motivated and metacognitively self-regulated students*” (adequate MMSR). In Table 4, the indicator means and standard deviations per profile are presented and compared to gain deeper understanding of how the interactions function. As seen in Table 4, the differences in motivational variables and metacognitive self-regulation were very large between profiles.

**Table 4:** Motivational and metacognitive self-regulation indicator means and standard deviations across profiles.

Indicator	Profile 1, M (SD)	Profile 2, M (SD)	Profile 3, M (SD)	Brown-Forsythe F	Partial η^2^
Self-efficacy	-0.90 (.91)	.13 (.76)	.84 (.55)	376.74***	.42
Mastery goal	-0.90 (.93)	.06 (.71)	.98 (.48)	478.26***	.47
Performance goal	-0.51 (1.05)	.07 (.92)	.47 (.81)	73.34***	.13
Intrinsic motivation	-1.00 (.73)	.05 (.68)	1.14 (.40)	863.56***	.60
Extrinsic motivation	-1.14 (.69)	.11 (.55)	1.16 (.31)	1326.21***	.71
Metacognitive self-regulation	-.91 (.80)	.08 (.77)	.94 (.60)	462.67***	.46

Note: All indicator means reached statistical significance at least at p < .05. The assumption of homogeneity of variances was rejected according to Levene’s test. M: Mean; SD: Standard deviation; Profile 1: Minimal MMSR; Profile 2: Adequate MMSR; Profile 3: Exceptional MMSR; ***p < .001.

### Variation in school language achievement across profiles

Next, the manual BCH method was deployed to explore whether profile membership was predicting achievement in language lessons adjusting for covariates. Given that ICC revealed 15% of the variance in school language achievement was explained by school-level factors, a fixed-effects estimation was implemented.

In the minimal MMSR profile, achievement was the lowest, *M* = 13.02, *SD* = 2.89, *p* < .001. The language grade in the adequate MMSR profile 2 was nearly two units higher with *M* = 16.05, *SD* = 2.18, *p* < .001, whereas mean achievement was the highest *M* = 18.01, *SD* = 2.13, *p* < .001, in the exceptional MMSR profile 3. Adjusting for covariates (Table 5), the mean language achievement between profiles 2 and 3 differed significantly with a large effect size, *Z =* -3.08, *p* = .002, Cohen’s *d* = .90. The mean achievement differed even more between profiles 1 and 2, *Z* = -2.69, *p* = .007, Cohen’s *d* = 1.23. Finally, the effect size of the mean achievement difference between profiles 1 and 3 was even larger, *Z* = -4.79, *p* = .000, Cohen’s *d* = 1.94.

**Table 5:** School language achievement differences across profiles and latent multinomial regressions predicting profile membership (fixed-effects estimation).

Achievement comparisons across profiles				
Profile contrast	Δ*μ*	*Z*	*p*-value	95% CI
P1-P2	-3.03	-2.69	.007	-5.23, -.82
P1-P3	-4.99	-4.79	.000	-7.03, -2.95
P2-P3	-1.96	-3.08	.002	-3.20, -.71
Latent Multinomial Regressions				
	*OR*	*Z*	*p*-value	95% CI
Profile 1				
Gender (male)	3.12	5.29	.000	2.05, 4.76
Age	1.45	2.35	.019	1.06, 1.99
SES	.80	-3.19	.001	.70, .92
Language at home (Greek)	.22	-2.95	.003	.08, .60
Profile 2				
Gender (male)	1.65	2.56	.010	1.12, 2.41
Age	1.18	1.14	.254	.89, 1.58
SES	.91	-1.51	.130	.81, 1.03
Language at home (Greek)	.55	-1.07	.282	.18, 1.63

Note: Profile 3 (Exceptional MMSR) was the reference profile; all regression coefficients represent differences in comparison to profile 3. P1: Minimal MMSR; P2: Adequate MMSR; P3: Exceptional MMSR; Δμ: Mean difference; OR: Odds ratio; 95% CI corresponds to the 95% confidence interval for the odds ratio and the mean differences, respectively.

Using the manual BCH method, multinomial logistic regressions were also estimated to explore the predictive effects of covariates on profile memberships. The BCH method revealed that being male predicted greater odds of membership in the minimal MMSR profile 1 (OR = 3.12, 95% CI 2.05, 4.76) and in the adequate MMSR profile 2 (OR = 1.65, 95% CI 1.12, 2.41) compared to the exceptional MMSR profile. Being older was related to greater odds of belonging to minimal MMSR profile 1 (OR = 1.45, 95% CI 1.06, 1.99) compared to the exceptional MMSR profile, confirming the decline in academic motivation in adolescence. Coming from a household with higher SES was related to lower likelihood of being in the minimal MMSR profile 1 (OR = .80, 95% CI = .70, .92). Speaking Greek language at home was associated only with lower odds of belonging to the minimal MMSR profile 1 (OR = .22, 95% CI .08, .60).

## Discussion

This study aimed to investigate the higher-order interactions between students’ language class motivation and metacognitive self-regulation guided by the MASRL model. Additionally, the study aimed to explore how these interactions were linked with students’ language class achievement in the Greek low-performance context. To achieve these objectives, LPA was used to mimic those higher-order interactions and link profile membership with school language class achievement. Hence, the present study contributes to the existing literature in multiple ways that will be described below.

### Profiles of motivational beliefs and metacognitive self-regulation: Exploring the form of the interaction hypothesis

The current LPA analyses revealed that Greek students’ motivated metacognitive self-regulation exhibited not only quantitative but also qualitative individual differences. Surprisingly, number of subgroups of students with similar motivational and metacognitive characteristics was smaller than initially hypothesised. Thus, H1 was rejected. In other words, although past studies indicated four or more emerging profiles [41–43,85], the current evidence suggests the existence of only three well-defined profiles of motivated metacognitive self-regulation.

In greater detail, the present findings indicated three profiles contained students who exhibited minimal, adequate, and exceptional scores on all indicators, respectively. The results revealed that 28.5% of the students had minimal scores in motivation and metacognitive self-regulation, whilst only 23.3% had exceptional scores on motivation and metacognitive self-regulation in school language lesson. 48.2% of the students had adequate scores across all variables. These findings are indicative of sixth-order interactions that occur in the same students [10,11]. Across the spectrum of different possible values of motivation and metacognitive self-regulation in the school language lesson, the interactions between self-efficacy, mastery and performance goal orientations, intrinsic and extrinsic motivation, and metacognitive self-regulation were synergistic, meaning that greater motivational beliefs were associated with greater planning, monitoring, and regulating cognitive processes in the language lesson.

Regarding the shape of the profiles, the initial hypotheses were that we would identify a profile including the interaction between high motivation and high metacognitive self-regulation, which was confirmed (H2). Another hypothesis was that a profile would emerge including low motivation and low metacognitive self-regulation, which was also confirmed (H3). Surprisingly, a profile including negative interaction between academic motivation and metacognitive self-regulation did not emerge. Thus, our H4 was rejected. This finding contradicts preceding evidence since the reviewed studies demonstrated that emerging profiles could comprise different levels of motivational beliefs and metacognitive strategies. For example, a study [13] found a profile of students whose motivational beliefs were low but their metacognitive knowledge was high. Similarly, another study [41] reported a profile of students with moderate scores on metacognitive strategies but low scores on motivational beliefs. Another study presented a profile with low self-regulation but high motivation [42]. An argument presented in some preceding studies [13] is that low levels of metacognitive self-regulation may be compensated by high motivation. However, the present study did not find any support for that argument.

It may be possible that profiles containing students with incompatible levels of motivation and metacognitive self-regulation or knowledge may indicate methodological differences such as the use of scales with different anchoring points which could induce variation in the sample variance of the measures, resulting, thus, in level-specific differences. Additionally, it is noted that profiles of students’ motivation and metacognitive self-regulation is dynamic and may exhibit significant differences across academic subjects [41] and developmental stages (e.g., adolescence vs. emerging adulthood) since different measurements and cognitive capabilities [86] could explain the inconclusive results across studies.

Additionally, this finding answers an important question since the MASRL model, which illustrates the potential for interactions between motivation and metacognition, does not specifically set what would be the directional nature of these interactions [1,46]. Indeed, MASRL outlines scenarios where the interaction between motivation self-beliefs, affect, and metacognition can be either positive or negative. An example of such a negative scenario would be a student that feels good about a school subject, has high self-efficacy that she can accomplish the task in the subject, and has a mastery- and performance-approach goal but is metacognitively inaccurate in judging the demands of a task in this subject (e.g., a test) and, thus, overestimates her capability. Nevertheless, the current findings urge us to reconsider how these particular interactions between motivational beliefs and metacognitive self-regulation should be theoretically modelled at the generic person-level since no such negative interactions emerged in this large sample of adolescent students. This may be specifically tied to the nature of the school language subject and the developmental period under study here but it, nevertheless, needs further research.

### Linking higher-order interactions between motivation and metacognition with school language achievement

Some person-centred studies have explored the effects of the interactive effects on students’ academic achievement. However, these studies focused on university/college course grades [41,43,87], performance in second language [12], or mathematics [88]. Nevertheless, it is contended that it is also important to address the issue of how these interactions are predictive of school language achievement, especially since language-related competencies, such as reading literacy, writing, comprehension, are increasingly important for society but many adolescents lag behind in their acquisition [89]. Additionally, especially in the Greek context, language achievement in adolescence has been found to be declining sharply across the years [19,90].

Hence, to address this important question of whether the interactions between motivational beliefs and metacognitive self-regulation were predictive of language achievement, students’ school language academic achievement was regressed on the latent categorical variable, adjusting for relevant covariates. The results indicated that those students with the lowest motivation and metacognitive self-regulation were the worst performing, whilst students with high motivation and metacognitive self-regulation were the most successful in school language courses. Although these findings do not indicate causality, they confirm, in line with theoretical evidence [3], that students need a high level of academic motivation to become highly metacognitively self-regulators. However, it remains to be seen whether motivation can compensate for failure in metacognitive self-regulation and how this may be linked with academic achievement.

### Covariate effects on motivational and metacognitive self-regulation profiles

In addition to the above, we accounted for the potential associations between a few control variables and motivational and metacognitive self-regulation profiles. The results of multinomial regressions revealed statistically significant gender differences in adolescent students’ motivational and metacognitive profiles, with male students having a greater probability of membership in the minimal and sufficient profiles. Thus, H6 was confirmed. Preceding person-centred studies did not account for students’ socio-economic (SES) background [13,42,88], we included this variable since it has been connected with achievement gaps [52]. The findings indicated that coming from a higher SES household was associated with lower likelihood of being less motivated and metacognitively self-regulated, confirming, thus, our seventh hypothesis (H7).

Although greater age has been associated with a decline in academic motivation [17,18], but an increase in the capability for metacognitive capabilities [15], we did not formulate clear theoretical assumptions given that ambiguity of the association between age and motivational and metacognitive profiles. Despite that, the regression results illustrated that being older was predictive only of greater likelihood of membership in the minimal motivation and metacognitive self-regulation profile. This confirmed our hypothesis suggesting that an association would emerge (H8). The observed negative association appears to be bilateral since the bivariate correlations suggested that both motivational variables and metacognitive self-regulation decreased with greater age. This may suggest that older students in this sample might be more careless and may not deploy maintain a sufficient level of metacognitive self-regulation in school language class, which may explain why there is a large percentage of low-performing students in PISA studies. Finally, students speaking Greek at home with their families had lower chances of being less motivated and metacognitively self-regulated. This suggests that speaking the school’s official language at home has a protective effect against becoming less motivated and metacognitively self-regulated, confirming, thus, our ninth hypothesis.

### Strengths and limitations

At this point, it is critical to reflect on some important strengths and limitations of this study. The large sample size, the low-performance context, the probability-based LPA, and the developmental stage of the participants can be considered amongst the strengths of this study. However, it ought to be noted that the sampling design is a convenience one and may not represent the whole Greek student population. Additionally, further measures indicative of SRL, such as cognitive strategies or task anxiety, were not included in this study, which limits the extent of the generalisations with respect to students’ SRL. Finally, the self-reported nature of the measures might have been influenced by social desirability.

### Policy, practice, and theoretical implications

In line with the current findings, teachers should facilitate the development of students’ motivational beliefs, values about school language achievement, and metacognitive self-regulatory strategies. Given the significant proportion of students with minimal motivation and metacognitive self-regulation, emphasis should be placed on the identification and targeted educational intervention for those students. Due to the low academic performance educational system [91], systematic metacognitive strategy teaching and enhancement of students’ language motivation are recommended to be integrated in the national curriculum in order to raise students’ school language achievement. Particular emphasis should be placed on maintaining male students’ motivation and metacognitive self-regulation because we found that male students had greater chances of belonging to the minimal MMSR profile. From an educational perspective, we also suggest providing individualised assistance to students from low-SES backgrounds and those who do not speak Greek at home to prevent them from becoming unmotivated, metacognitively unregulated, and, subsequently, low-performers.

From a theoretical viewpoint, the present study contributes to existing social-cognitive models of SRL [1,2] that typically do not explicate what would be the consequences of a failure either in metacognitive self-regulation or self-motivation for academic achievement. That is, the current study examined through a person-centred perspective whether low functioning in either metacognitive self-regulation or motivation could be compensated by higher functioning in the other domain. The results suggest that current SRL models should clearly explicate that students’ motivation and metacognitive self-regulation are synergistically interacting across all levels of achievement. Additionally, the present findings challenge existing knowledge by suggesting that there is a limited number of subgroups of students with individual differences in motivation and metacognitive self-regulation.

## Supporting information

S1 Table Academic self-efficacy in school language class. (DOCX)

S2 Table Mastery Goals- Intrinsic Goals. (DOCX)

S3 Table Performance Goals- Extrinsic Goals. (DOCX)

S4 Table Intrinsic motivation. (DOCX)

S5 Table Extrinsic motivation. (DOCX)

S6 Table Metacognitive Self-regulation. (DOCX)

S7 Table Confirmatory factor analysis of the mastery scale. (DOCX)

S8 Table Confirmatory factor analysis of the performance goal scale. (DOCX)

S9 Table Confirmatory factor analysis of the self-efficacy. (DOCX)

S10 Table Confirmatory factor analysis of the intrinsic and extrinsic motivation. (DOCX)

S11 Table Confirmatory factor analysis of the metacognitive self-regulation scale. (DOCX)

S1 Text Outliers and missing data analysis. (DOCX)

S2 Text Further measures. (DOCX)
