# Supplementary material for: Person-centred study on higher-order interactions between students’ motivational beliefs and metacognitive self-regulation: Links with school language achievement
Source: PLoS One. 2023 Oct 4;18(10):e0289367. doi: 10.1371/journal.pone.0289367 (PMC10550156; doi:10.1371/journal.pone.0289367)
Supplement: S1 Table — (DOCX) [file pone.0289367.s001.docx]

**S1 Table. Academic self-efficacy in school language class**

| 1. Compared with other students in this **class** I expect to do well |
| --- |
| 1. I'm certain I can understand the ideas taught in this course |
| 1. I expect to do very well in this class |
| 1. Compared with others in this class, I think I'm a good student |
| 1. I am sure I can do an excellent job on the problems and tasks assigned for this class |
| 1. I think I will receive a good grade in this class |
| 1. My study skills are excellent compared with others in this class |
| 1. Compared with other students in this class I think I know a great deal about the subject |
| 1. I know that I will be able to learn the material for this class |

Class was substituted with modern Greek language class throughout the questionnaire. The word “course” was changed to modern Greek language class throughout for consistency purposes.
