# Supplementary material for: Person-centred study on higher-order interactions between students’ motivational beliefs and metacognitive self-regulation: Links with school language achievement
Source: PLoS One. 2023 Oct 4;18(10):e0289367. doi: 10.1371/journal.pone.0289367 (PMC10550156; doi:10.1371/journal.pone.0289367)
Supplement: S2 Table — (DOCX) [file pone.0289367.s002.docx]

**S2 Table. Mastery Goals- Intrinsic Goals**

| 1. In a class like this, I prefer course material that really challenges me so I can learn new things |
| --- |
| 1. In a class like this, I prefer course material that arouses my curiosity, even if it is difficult to learn |
| 1. The most satisfying thing for me in this course is trying to understand the content as thoroughly as possible |
| 1. When I have the opportunity in this class, I choose course assignments that I can learn from even if they don’t guarantee a good grade |
