# Supplementary material for: Person-centred study on higher-order interactions between students’ motivational beliefs and metacognitive self-regulation: Links with school language achievement
Source: PLoS One. 2023 Oct 4;18(10):e0289367. doi: 10.1371/journal.pone.0289367 (PMC10550156; doi:10.1371/journal.pone.0289367)
Supplement: S3 Table — (DOCX) [file pone.0289367.s003.docx]

**S3 Table. Performance Goals- Extrinsic Goals**

| 1. Getting a good grade in this class is the most satisfying thing for me right now |
| --- |
| 1. The most important thing for me right now is improving my overall grade point average, so my main concern in this class is getting a good grade |
| 1. If I can, I want to get better grades in this class than most of the other students |
| 1. I want to do well in this class because it is important to show my ability to my family, friends, future employer, or others. |
