# Supplementary material for: Person-centred study on higher-order interactions between students’ motivational beliefs and metacognitive self-regulation: Links with school language achievement
Source: PLoS One. 2023 Oct 4;18(10):e0289367. doi: 10.1371/journal.pone.0289367 (PMC10550156; doi:10.1371/journal.pone.0289367)
Supplement: S4 Table — (DOCX) [file pone.0289367.s004.docx]

**S4 Table. Intrinsic motivation**

| 1. I am very interested in the content area of this course |
| --- |
| 1. I like the subject matter of this course |
