# Supplementary material for: Person-centred study on higher-order interactions between students’ motivational beliefs and metacognitive self-regulation: Links with school language achievement
Source: PLoS One. 2023 Oct 4;18(10):e0289367. doi: 10.1371/journal.pone.0289367 (PMC10550156; doi:10.1371/journal.pone.0289367)
Supplement: S5 Table — (DOCX) [file pone.0289367.s005.docx]

**S5 Table. Extrinsic motivation**

| 1. I think I will be able to use what I learn in this class in other courses |
| --- |
| 1. It is important for me to learn the course material in this class |
| 1. I think the course material in this class is useful for me to learn |
| 1. Understanding the subject matter of this course is important for me |
