# Supplementary material for: Person-centred study on higher-order interactions between students’ motivational beliefs and metacognitive self-regulation: Links with school language achievement
Source: PLoS One. 2023 Oct 4;18(10):e0289367. doi: 10.1371/journal.pone.0289367 (PMC10550156; doi:10.1371/journal.pone.0289367)
Supplement: S6 Table — (DOCX) [file pone.0289367.s006.docx]

**S6 Table. Metacognitive self-regulation**

| 1. I ask myself questions to make sure I know the material I have been studying |
| --- |
| 1. I work on practice exercises and answer end of chapter questions even when I don't have to. |
| 1. Even when study materials are dull and uninteresting, I keep working until I finish |
| 1. Before I begin studying, I think about the things I will need to do to learn |
| 1. When I'm reading, I stop once in a while and go over what I have read |
| 1. I work hard to get a good grade even when I don't like a class |

Note: Three items from the original scale were dropped from the analyses due to low latent factor loadings even after reverse-scoring due to negative item wordings.
