# Supplementary material for: Person-centred study on higher-order interactions between students’ motivational beliefs and metacognitive self-regulation: Links with school language achievement
Source: PLoS One. 2023 Oct 4;18(10):e0289367. doi: 10.1371/journal.pone.0289367 (PMC10550156; doi:10.1371/journal.pone.0289367)
Supplement: S7 Table — (DOCX) [file pone.0289367.s007.docx]

**S9 Table. Confirmatory factor analysis of the mastery goal orientation scale**

| Item | Factor Loading |
| --- | --- |
| Item 1 | .502*** |
| Item 2 | .490*** |
| Item 3 | .793*** |
| Item 4 | .457*** |
| Inter-item residual correlation | Coefficient |
| Item 2 WITH Item 1 | .365*** |

****p<.001*
