# Supplementary material for: Person-centred study on higher-order interactions between students’ motivational beliefs and metacognitive self-regulation: Links with school language achievement
Source: PLoS One. 2023 Oct 4;18(10):e0289367. doi: 10.1371/journal.pone.0289367 (PMC10550156; doi:10.1371/journal.pone.0289367)
Supplement: S8 Table — (DOCX) [file pone.0289367.s008.docx]

**S10 Table. Confirmatory factor analysis of the performance goal orientation scale**

| Item | Factor Loading |
| --- | --- |
| Item 1 | .713*** |
| Item 2 | .718*** |
| Item 3 | .565*** |
| Item 4 | .560*** |

****p<.001*
