# Supplementary material for: Person-centred study on higher-order interactions between students’ motivational beliefs and metacognitive self-regulation: Links with school language achievement
Source: PLoS One. 2023 Oct 4;18(10):e0289367. doi: 10.1371/journal.pone.0289367 (PMC10550156; doi:10.1371/journal.pone.0289367)
Supplement: S9 Table — (DOCX) [file pone.0289367.s009.docx]

**S11 Table. Confirmatory factor analysis of the self-efficacy in school language lesson scale**

| Item | Factor Loading |
| --- | --- |
| Item 1 | .648*** |
| Item 2 | .654*** |
| Item 3 | .789*** |
| Item 4 | .794*** |
| Item 5 | .703*** |
| Item 6 | .777*** |
| Item 7 | .783*** |
| Item 8 | .716*** |
| Item 9 | .654*** |

****p<.001*
