# Supplementary material for: Person-centred study on higher-order interactions between students’ motivational beliefs and metacognitive self-regulation: Links with school language achievement
Source: PLoS One. 2023 Oct 4;18(10):e0289367. doi: 10.1371/journal.pone.0289367 (PMC10550156; doi:10.1371/journal.pone.0289367)
Supplement: S10 Table — (DOCX) [file pone.0289367.s010.docx]

**S12 Table. Confirmatory factor analysis of the intrinsic and extrinsic motivation scale (two-factor solution)**

| Item | Factor Loading |
| --- | --- |
| Intrinsic Factor |  |
| Item 1 | .867*** |
| Item 2 | .803*** |
| Extrinsic Factor |  |
| Item 1 | .723*** |
| Item 2 | .769*** |
| Item 3 | .833*** |
| Item 4 | .798*** |
| Latent Factor Correlation | Coefficient |
|  | .914*** |

****p<.001*
