# Supplementary material for: Person-centred study on higher-order interactions between students’ motivational beliefs and metacognitive self-regulation: Links with school language achievement
Source: PLoS One. 2023 Oct 4;18(10):e0289367. doi: 10.1371/journal.pone.0289367 (PMC10550156; doi:10.1371/journal.pone.0289367)
Supplement: S11 Table — (DOCX) [file pone.0289367.s011.docx]

**S13 Table. Confirmatory factor analysis of the metacognitive self-regulation scale**

| Item | Factor Loading |
| --- | --- |
| Item 1 | .737*** |
| Item 2 | .606*** |
| Item 3 | .615*** |
| Item 4 | .657*** |
| Item 5 | .441*** |
| Item 6 | .493*** |
| Inter-item residual correlation | Coefficient |
| Item 4 WITH Item 6 | .306*** |

****p<.001*
