# Supplementary material for: Person-centred study on higher-order interactions between students’ motivational beliefs and metacognitive self-regulation: Links with school language achievement
Source: PLoS One. 2023 Oct 4;18(10):e0289367. doi: 10.1371/journal.pone.0289367 (PMC10550156; doi:10.1371/journal.pone.0289367)
Supplement: S1 Text — (DOCX) [file pone.0289367.s012.docx]

**S8 Text. Outliers and missing data analysis**

Multivariate outliers were examined through the LPA analysis using the influence statistic in M*plus*. All influence values were below 1 ranging between .89>INF>.001. Thus, no potential outliers were identified for the LPA analysis (Tabachnick & Fidell, 2012). To explore the missing data mechanism, missing data patterns were subjected to Little’s MCAR (missing completely at random test) test. Little’s MCAR reached statistical significance, MCAR *χ*^2^(1009)= 1292.123, *p*<.001. Thus, the missing values were not missing completely at random. Next, we tested whether the hypothesis of missing at random data conditionally on covariates (i.e., sex, class, age, SES, and home language). The CDF *χ*^2^(5304)= 2419.121, *p*>.05, indicated that the missing data patterns were related to covariates. Hence, we could analyse the data using full-information maximum likelihood estimation.
