# Supplementary material for: Person-centred study on higher-order interactions between students’ motivational beliefs and metacognitive self-regulation: Links with school language achievement
Source: PLoS One. 2023 Oct 4;18(10):e0289367. doi: 10.1371/journal.pone.0289367 (PMC10550156; doi:10.1371/journal.pone.0289367)
Supplement: S2 Text — (DOCX) [file pone.0289367.s013.docx]

**S7 Supporting Information. Further measures**

| **Achievement Score in Language**   - Please circle your most recent grade in modern Greek language class   1 2 3 4 5 6 7 8 9 10 11 12 13 14 15 16 17 18 19 20  **Demographic Information**   - How would you describe your gender?   Male □ (1) Female □ (0)   - What school grade are you currently in?   A’ Gymnasium □ (0) B’ Gymnasium □ (1) C’ Gymnasium □ (2) A’ Lyceum □ (3)   - How old are you?   13 □ 14 □ 15 □ 16 □   - Imagine that this ladder pictures how GREEK society is set up. At the top of the ladder are the people who are the best off — they have the most money, the highest amount of schooling, and the jobs that bring the most respect. At the bottom are people who are the worst off — they have the least money, little or no education, no job, or jobs that no one wants or respects. Now think about your family. Please tell us where you think your family would be on this ladder. Mark the rung that best represents where your family would be on this ladder.   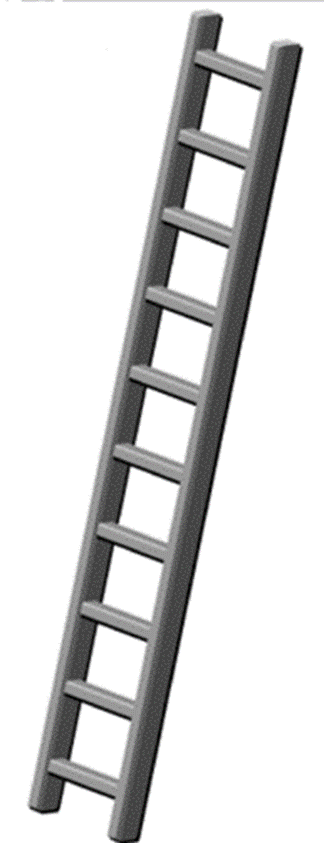   - Is Greek the language you speak at home with your family?   Yes □ (1) No □ (0) |
| --- |
